# Indents

**Published:** 1912-11-15

**Authors:** 


### INDENTS.

☞Brief Articles of Technical or Scientific Value will receive notice and comment. Contributions invited.

*Neuralgia.* A later method of treating neuralgia is the injection of 3 minims of 90-percent. alcohol into the branch of the nerve. The fluid is thrown through a long and delicate needle into the nerve at its deepest accessible point, at the exit of the skull. Severe pain lasting a few months often follows the injection. Frequently the area of skin becomes anesthetic and remains so for months. Success has been reported by several observers. —*Clinical Medicine.*

*Oral Prophylaxis.* In a paper read at Watertown, N. Y., Alfred R. Starr, D.D.S., of New York, said:

"*At the present time we hear a great deal about oral prophylaxis and the necessity for educating the masses in that subject.*

"*There is absolutely no question in regard to the importance of the subject and the desirability of impressing upon the laity the necessity for practising its precepts, but there is a lurking suspicion in the minds of many, that some of those who are most active in spreading its propaganda are not actuated solely by altruistic motives.*

"*There are some who aim to specialize in the practice of oral prophylaxis, but while I am confident of the advantages of specialization in most cases, I do not think the time is yet ripe for specialization in this branch, unless it be included as a prerogative for those who specialize in the treatment of pyorrhea. It is so important and so fundamental a principle of dental practice that it should be an integral part of the work of every practitioner.*"

He ends with some good advice in saying, "*It should be an integral part of the work of every practitioner.*"

This has been a fact ever since we have realized what a blessing oral prophylaxis is, and every dental society, Local, State and National, has been bombarded with papers urging the dentist to follow the good advice, but have they? Only a small per cent. There are many reasons for this neglect. The ones most often mentioned is the inability, or fear, to charge prices commensurate with the labor necessary to properly do this class of work; second, the reason given by a number of "Country Dentists" was "they admitted oral prophylaxis would eliminate fifty per cent or more of future repair work, and were not fools enough to ruin their own business." They claimed a city dentist, with his large population to draw from, would not be affected this way. Such a flimsy excuse might fill a long felt want with some, but while a true one in several instances is of too reflecting a character to be applied to many. The average dentist does not enjoy cleaning teeth and does not clean them. It is a custom with many to brush off labial surfaces and make no charge. The patients imagine their teeth are cleaned, the same as the small boy who smears a wet towel on his face and seeing a clean spot resulting thinks he is washed. The time for the Oral Prophylaxis Specialist should have arrived and probably should be in combination with the treatment of pyorrhea, and it seems as if every large city should support one or more specialists, and undoubtedly such would be the case if those who are qualified could believe they would get the support of those who either cannot or will not perform these needed operations.—J. P. Root in *Western Journal.*

*Iodine in Mouthwash.* Carles (*Gaz. hebd. des science med.*, Bordeaux), advocates the use of iodine as a mouth-wash, especially in cases of foul breath due to dental caries. One gramme of potassium iodide should be added to 20 grm. of iodine tincture, and of this mixture from one to three drops should be thrown into a quarter of a glass of warm water. With this solution the mouth should be carefully rinsed. The hotter the water the more drops it will carry. If iodine tincture is used alone the iodine is separated out by the water, and clinging to the mucous membrane causes a lasting and disagreeable taste. Potassium iodide keeps the iodine in solution, and the taste becomes mild and pleasant. The drug penetrates to all the crevices of the mouth, and acts as a deodorant and an antiseptic. The lotion is to be recommended as a prophylactic to those with sound teeth, and the writer is convinced that by employing it regularly, especially at bedtime, caries can be prevented. The teeth are not permanently stained by the dilution mentioned.

*Opening Teeth Devitalized with Arsenic.*

Opening teeth devitalized with arsenic—especially molars—with large round burs—is to be deprecated, as causing the patient much unnecessary pain. Such is the practice usually taught, but the teaching is evidently wrong or wrongly applied.

The author's experience has been that after the application of minute quantities of arsenic, for ten days or even longer, the canal portions of the pulp are still vital, and will respond keenly to any pressure produced by opening into the pulp chmaber. If a large bur is used for this purpose, and particularly a large round one, a certain amount of rootward pressure is unavoidable. The opening of the pulp chamber and the removal of the contents can, however, be accomplished in the manner described, without the slightest twinge of pain.

The majority of teeth to which arsenic has been applied, contain a slight opening in the roof of the pulp chamber, exposing the pulp, and close to which the arsenic is usually applied. Remove the fibre or paste with an excavator, and gently enlarge this opening with a veiy small inverted cone bur, gradually working the edge of the bur beneath the roof of the pulp chamber, drawing it all the time towards the masticating surface of the tooth. Any pressure upon the pulp is thus avoided. A larger inverted cone may then be substituted, and when the entire roof has been removed, and not before, a large round bur may be resorted to, as a good view of the pulp chamber is now secured, and its contents may be quite painlessly removed, ample room being provided to manipulate a good-sized round bur, without causing pressure upon any portion of the pulp within the canals that may still be vital.—Dr. Paul Clipsham in *Commonwealth Dental Review.*

*Silicate Cements, Packing.* Important as are the directions sent out with the various makes of silicate cements to insure a thorough and clean mix, the permanency and usefulness of these cements will depend quite as much upon the care used in packing them into the cavity. They have not the body possessed by the oxychlorid and phosphate cements, the packing tools push through them instead, pressing them into place, and there is a tendency to merely plaster over the cavity instead of thoroughly filling it. An examination of fillings which have "vacated" will usually disclose that they do not present a sharp impression of the cavity. We, of course, assign the untoward result to the cement "contracting," while in all probability, as a matter of fact, the real retaining elements of cavity preparation were not reached by the cement. The writer has adopted the plan of partly filling the cavity with cement, and then pressing into it firmly a pellet of clean non-absorbent cotton (raw cotton), removing the cotton and immediately completing the filling. The cotton presses a film of cement into close contact with the cavity walls and this seems to facilitate further additions of cement.—W. H. T., —*The Dental Brief.*

*Cosmetology.* A Cosmetologist, while he may not know it is one who practices cosmetology. Cosmetology pertains to the cosmetic care of the body. The cosmetic care of the body pertains to its beautifying and improving.

Hence a dentist who practices his profession as he should is a cosmetologist. Now that we know what we are, or should be, let us see where cosmetics interest us as dentists. Ordinarily when mentioning the word cosmetics, one thinks only of the cold cream and powder our lady friends use to prove, improve or disprove their complexions, but it is dental cosmetology we are interested in, for by following its practices we are assisting nature with art, in making the female race more beautiful.

When the "other fellow's" patient comes to us, and he has placed gold crowns where porcelain should be, we can make a marked improvement by removing them and replacing with porcelain, and probably there is more demand for this cosmetic dental operation than any other, more especially in replacing gold bicuspids, the insertion of which for men is at times excusable, but rarely so for women. Belasco, the playwright and theatrical manager, appreciates the horrors of gold crowns to such an extent as to issue a sweeping order that no actor or actress could appear in his plays who wore a gold crown, visible to the audience, and to anyone who has been fascinated by the limelight shining on and magnifying the unesthetic crown of a stage hero or villain, can appreciate this order.

Nature often produces one or more incisors a third longer than their companions, resulting in a carnivorus aspect not necessary. A stone and sandpaper disk will soon remedy this defect. Yourself, or competitor, often leave amalgam fillings with over-lapping edges, or an excess remains in approximal space, or, as is generally the case with old fillings, there is a bulging in case of amalgam, or a chipping with gold. Again, the stones and disks will greatly improve conditions.

There are many different defect due to nature or man. We can by practicing cosmetology improve our patient's appearance and our reputations, and possibly by perseverance acquire the title of a specialist of Prophylactic Cosmetology.—*Western Journal.*

*Influence of Mastication on the Condition of the Teeth.* Investigations on the children in the town of Koetzling in Bavaria, shows that of those who eat hard bread, the percentage with bad teeth was 6.9; of those who eat both hard and soft bread, 8.2; of those eating only soft bread, 10.5 In the town of Ihringen (Baden) the percentages before and after the introduction of soft bread were as follows: In 1894, when only hard bread was eaten, 12.4 per cent.; in 1897, just after soft bread had been introduced, 12.9 per cent.; and in 1901, when most of the bread consumed was soft, 20.9 per cent.—*Dental Record.*

*Obtaining Smooth Palatal Surface in Vulcanite Dentures.* In order to prevent plaster from sticking to a rubber plate and to obtain a smooth palatal surface the plaster model is coated with a layer of collodion mixed with powdered aluminum. In this way a smooth, metal-like surface is obtained on the model, to which the rubber does not adhere after vulcanizing.—*Schweizerische Vierteljahrsschrift fuer Zahnheilkunde.*

*Restoring Clogged Carborundum Wheels.* In order to restore clogged carborundum wheels which have become dull and useless after polishing amalgam fillings, the wheels are immersed into a fifty per cent. nitric acid solution for two or three hours, and then transferred into a bath of concentrated sodium bicarbonate solution, in which they are left until the acid absorbed by the wheels has been neutralized.—*Revue Trimestrielle Belge de Stomatologie.*

*Take Your Own Medicine.* In discussing the good and bad qualities of our patients, we often hear the expression, "He is a baby," or one a little stronger, such as, "He is a d——— F." While the definition may truthfully apply to some, the chances are we are mistaken in our judgment and diagnosis, especially so, if the dentist is fortunate enough to possess a set of sound teeth, or is not over susceptible to pain.

It is an impossibility for those who have not suffered the excruciating pain resulting from the proper preparation of a cavity, or the more than excruciation that follows the scaler when removing deep deposits in treating pyorrhea to have the remotest idea of the suffering one enjoys, and of course this enjoyment varies in degree according to the humanitarian disposition of the dentist. There is a vast difference in our sympathetic natures, which is of vital importance to our patients, although the majority of them anticipate pain and misery, and as a rule their anticipations are realized.

There is a minimum and a maximum of pain that can be inflicted, and the minimum is more than apt to come from the hands of a dentist who has suffered the maximum for, unless such suffering has been his, he can never have the remotest conception of the sensation following a red hot bur, or the trail of a scaler, hence the title "Take Your Own Medicine" is one that should be literally followed. It would be a more cheerful outlook for the public if a law could be passed whereby at stated intervals we should have a sensitive cavity prepared and a still wiser prophylactic measure would be for the Association of Faculties to demand as a preliminary requirement for entrance to a dental college that every student should possess teeth of a supersensitive structure.

If these statements are not believed, spend five hours in the chair of one of your fellow criminals, as did the writer, then meditate on the results of taking your own medicine. —*Western Journal.*

*Some Systemic Causes of Pyorrhea.* The causes of pyorrhea are commonly classed as local and systemic, the local causes being calculus and its consequent unhealthy environment. Of this class it is not the purpose of this paper to treat. They are found in patients usually under forty years of age and yield readily to local treatment, which is the only kind the dentist can give, and constitute the large and growing list of "cures" reported by the pyorrhea specialists.

The systemic causes may be subdivided for present purposes into two classes; first, those having for their origin ptyalisim, scurvy, Bright's disease and the gouty rheumatic diathesis and perhaps others, and, second, those having for their intial cause a weakening of the vitality due to advancing years with a consequent impairment of the capillary circulation, and the lowered power of resistance to disease.

How far the systemic diseases just mentioned are responsible for pyorrhea it is difficult to say, but they must be a large factor in the problem. We know that mercury will cause the loosening and falling out of the teeth; so will the scorbutic taint. The rheumatic or gouty diathesis has been credited as a large contributing factor, though many pyorrhetics of the worst type declare they have never had it, and many rheumatics do not have pyorrhea. Dr. Rhein has, I believe, assigned Bright's disease as a frequent cause. The diseases themselves are often obscure and may be unsuspected and denied, or intentionally concealed by the patient. At any rate they are obviously beyond the reach of the dentist who can only treat the local conditions —in other words, "treat the symptoms." Of course the hidden cause remains and, therefore, only a limited and temporary improvement of local conditions can be obtained in this class of cases.

*Age an Etiological Factor in Pyorrhea.*

The second, and by far the largest, contributing factor of the systemic causes of pyorrhea is the lessening of vitality due to age. That advancing years does bring a lessening of vitality cannot be denied. This is shown in many ways; the individual yields more easily to the attacks of disease; does not respond so promptly to treatment and recovers more slowly; the circulation is feebler and the powers of assimilation and elimination are impaired. The tissues partake of the general impairment and are subject to diseases that do not attack them in youth. The dermoid tissues especially, of which the teeth are a part, show an impairment of the capillary circulation; cancer becomes more frequent, and peripheral poverty is shown to the extent of finger nails wasting from inanition, the falling out of the hair, and the phenomenon we know as pyorrhea. Lack of vitality is for these the initial lesion and the presence of calculus but the secondary cause. This explains two phenomena that cannot be explained in any other way. When there is no impairment of vitality we find calculus, but no pyorrhea. When there is impairment of vitality but no calculus we find pyorrhea without the calculus.

*Philosophy, Science and Medicine.* "No philosophy evolved from the inner consciousness of man has ever done man half the good that has been secured to him by the discovery of the agents of infection. In fact, no discovery in science has failed to better the lot of man." With these significant words Prof. V. C. Vaughan of the University of Michigan replies, in an admirable address[4] that merits wide distribution, to the strictures of those modern writers who charge science with being essentially materialistic in its aims and scope. These philosophers, evolving what they assume to be an exalted idealism out of their inner self, are all too frequently in apparent ignorance of the real contributions which science has made alike to knowledge and to human happiness. Confident that the "inner life" contributes some superior sort of normal worth to the individual, they fail to grasp the significance of the forces about us and the biologic tendencies within us which determine in largest measure what constitutes human welfare and social and racial betterment.

Scientific men (and these necessarily include medical

4Vaughan, V. C.: The Philosophy of the Scientist, Science, Aug. 23, p. 225.

men) build their idealism solidly up from the ground; they do not suspend it precariously from star-beams. "The foundation-stone of my philosophy," writes Professor Vaughan, "is the doctrine of evolution." He points out that we deal here not with inscrutable forces which man cannot know, cannot modify, cannot study, but rather with potent factors that call for the best effort in behalf of the race. What higher incentive can there be, for example, to keep one's self clean morally and physically than the facts derived from the study of heredity? And when the added undeniable influence of environment in the modification and improvement of the species is taken into account, it is apparent that every effort to improve the conditions under which men live is based on motives quite as dignified and worth while as the musings of the philosopher who claims to cultivate the good and beautiful for its own sake.

It is timely to refute the charges of materialism which are now and then preferred against medicine. As Vaughan points out, our knowledge of the spread of infection is the strongest factor in the social movements of the day. Disease still takes a heavy toll. Ignorance and deep-seated prejudice still are often found retarding progress in unexpected quarters. The philosophy of science concerns itself with this world and this life and reaches every condition. It is a truth which will bear insistence and repitition that the dicta of science are a high, noble and powerful incentive to righteousness. "To widen the domain of knowledge, be it ever so little, to abate disease, to lessen pain and suffering, to decrease the burden of poverty, to brighten and ennoble the lives of others, to harness the forces of Nature and make them subservient to man's will and contributory to his happiness . . . . to make man more considerate of his fellow, to apprciate and perform his duties—these are some of the things that science has done and is doing. —*Journal A. M. A.*

